# Hereditary angioedema (HAE) in Belgium: results from a national survey

**DOI:** 10.3389/falgy.2023.1143897

**Published:** 2023-05-26

**Authors:** MM Van der Poorten, R Schrijvers, C Hermans, M Bartiaux, F Haerynck, H Lapeere, M Moutschen, O Michel, V Sabato, DG Ebo, AL Van Gasse

**Affiliations:** ^1^ Faculty of Medicine and Health Science, Department of Immunology – Allergology – Rheumatology, Antwerp University Hospital and the Infla-Med Centre of Excellence, University of Antwerp, Antwerp, Belgium; ^2^ Faculty of Medicine and Health Science, Department of Paediatrics, University of Antwerp, Antwerp University Hospital, Antwerp, Belgium; ^3^ Faculty of Medicine, Department of Microbiology, Immunology and Transplantation, Allergy and Clinical Immunology Research Group, KU Leuven, Leuven, Belgium; ^4^ Faculty of Medicine and Health Science, Department of Adult Haematology, Saint-Luc University Hospital, Brussels, Belgium; ^5^ Faculty of Medicine and Health Science, Department of Urgent Medicine, Hôpital Sient-Pierre, Brussels, Belgium; ^6^ Faculty of Medicine and Health Science, Department of Internal Medicine and Paediatrics, Ghent University Hospital, Ghent, Belgium; ^7^ Faculty of Medicine and Health Science, Department of Dermatology, Ghent University Hospital, Ghent, Belgium; ^8^ Faculty of Medicine and Health Science, Department of Internal Medicine and Infectious Diseases, C.H.U. de Liège - Site du Sart Tilman, Liège, Belgium; ^9^ Faculty of Medicine, Department of Immunology and Allergology, C.H.U. Brugmann, Brussels, Belgium

**Keywords:** hereditary angioedema, epidemiology, nationwide, Belgium, diagnosis, rare disease

## Abstract

**Background:**

Hereditary angioedema (HAE) is a rare heritable disorder that is characterized by recurrent, circumscribed, nonpitting, nonpruritic, often painful subepithelial swellings of sudden unpredictable onset that generally fade during 48–72 h. Epidemiological data of hereditary angioedema patients in Belgium is lacking.

**Methods:**

We set up a nation-wide, multicentric study involving the 8 Belgian hospitals known to follow-up patients with Type I and II HAE. All Belgium HAE patients were asked to fill out questionnaires that mainly covered demographic data, family history, and detailed information about diagnosis, treatment and burden of their Type I and II HAE.

**Results:**

112 patients with type I or type II HAE could be included. Median delay between first symptoms and diagnosis was 7 years. 51% of patients had experienced pharyngeal or tongue swelling and 78% had experienced abdominal symptoms, both known to cause an important reduction in quality of life. 60% of symptomatic patients reported to receive long term prophylactic treatment. Human plasma-derived C1-esterase inhibitor concentrate was used by 56.3% of patients. 16.7% and 27.1% of patients used a 17-α-alkylated androgen and tranexamic acid as long term prophylactic therapy.

**Conclusions:**

We present the first nation-wide epidemiological study regarding HAE in Belgium. Our data show that the morbidity of HAE is not to be underestimated. Knowledge and dissemination of this data is critical in raising awareness, encouraging development of therapies and optimising nationwide management.

## Introduction

Hereditary angioedema (HAE) is a rare heritable disorder that is characterized by recurrent, circumscribed, nonpitting, nonpruritic, often painful subepithelial swellings of sudden unpredictable onset that generally fade during 48–96 h.

Patients with HAE experience angioedema because of a defective control of the plasma kinin forming cascade (1). Type I and II HAE are characterized by a deficiency in the complement factor 1 esterase inhibitor (C1-INH) and are henceforth designated as C1-INH-HAE. Type I is characterized by low serum levels of C1-INH. In type II, serum levels of C1-INH are normal or even elevated, but the protein is dysfunctional. As recommended in the recently updated WAO/EAACI guidelines (2) and in an international consensus report on HAE in children (3), diagnosis of C1-INH-HAE should start with an evocative personal and familial history complemented with a measurement of complement factor C4 (that is usually decreased) combined with the demonstration of a deficient C1-INH function. To distinguish between type I and type II HAE, additional antigenic quantification of C1-INH could be performed (4, 5).

HAE with normal C1-INH is clinically indistinguishable from type I and II C1-INH-HAE. However, these patients show normal values for C4, C1-INH function and C1-INH quantification. Normal C1-INH HAE will not be the focus of this study. For a state of the art on the pathophysiology of normal C1-INH-HAE, the reader is referred elsewhere (2, 6, 7).

The prevalence of C1-INH-HAE is often estimated in research, but substantial epidemiological studies remain scarce. Population-based epidemiological studies have been conducted in various European countries (8–18), while data regarding the Belgian population remains lacking. This while knowledge of epidemiological data is critical in raising awareness, encouraging development of therapies and optimising management.

Here, we describe epidemiological and clinical data obtained *via* a standardized questionnaire that was completed by 112 Belgian type I and II C1-INH-HAE patients.

## Materials and methods

We set up a nation-wide, multicentric study involving the 8 Belgian hospitals known to follow-up patients with C1-INH-HAE. The centers included were: (*information removed due to blinding of the manuscript)*. The study was approved by the local committees of all the participating centers (registration number BE300201734006). Patients or their caregivers provided a written informed consent according to the declaration of Helsinki. Principal investigators and coinvestigators of all centers contributed to the preparation and standardization of the questionnaires that can be found in the repository file. Briefly, criteria for inclusion were a diagnosis of type I or II C1-INH-HAE. Diagnosis of C1-INH HAE type I and II was based upon a decreased C4 and a decreased C1-inhibitor function or a decreased C1-inhibitor antigenic quantification in case of family history (2).

All participants who agreed on participation were included after they completed an electronic questionnaire (see repository file). The questionnaire was encrypted and anonymous, with a decoding table only accessible by the treating physician.

Questionnaires mainly covered demographic data, family history, and detailed information about diagnosis, treatment and burden of their C1-INH-HAE. Duplicates were avoided by checking the patients’ medical history in their electronic medical file upon first presentation. This includes previous contacts at other reference centers. Moreover, before analysis of data, we checked for potential duplicates by checking dates of birth combined with the patient sex. No potential duplicates were identified.

Next, we performed descriptive statistics. Data are presented as numbers, percentages and median with range.

## Results

### Inclusion

**Figure 1:**
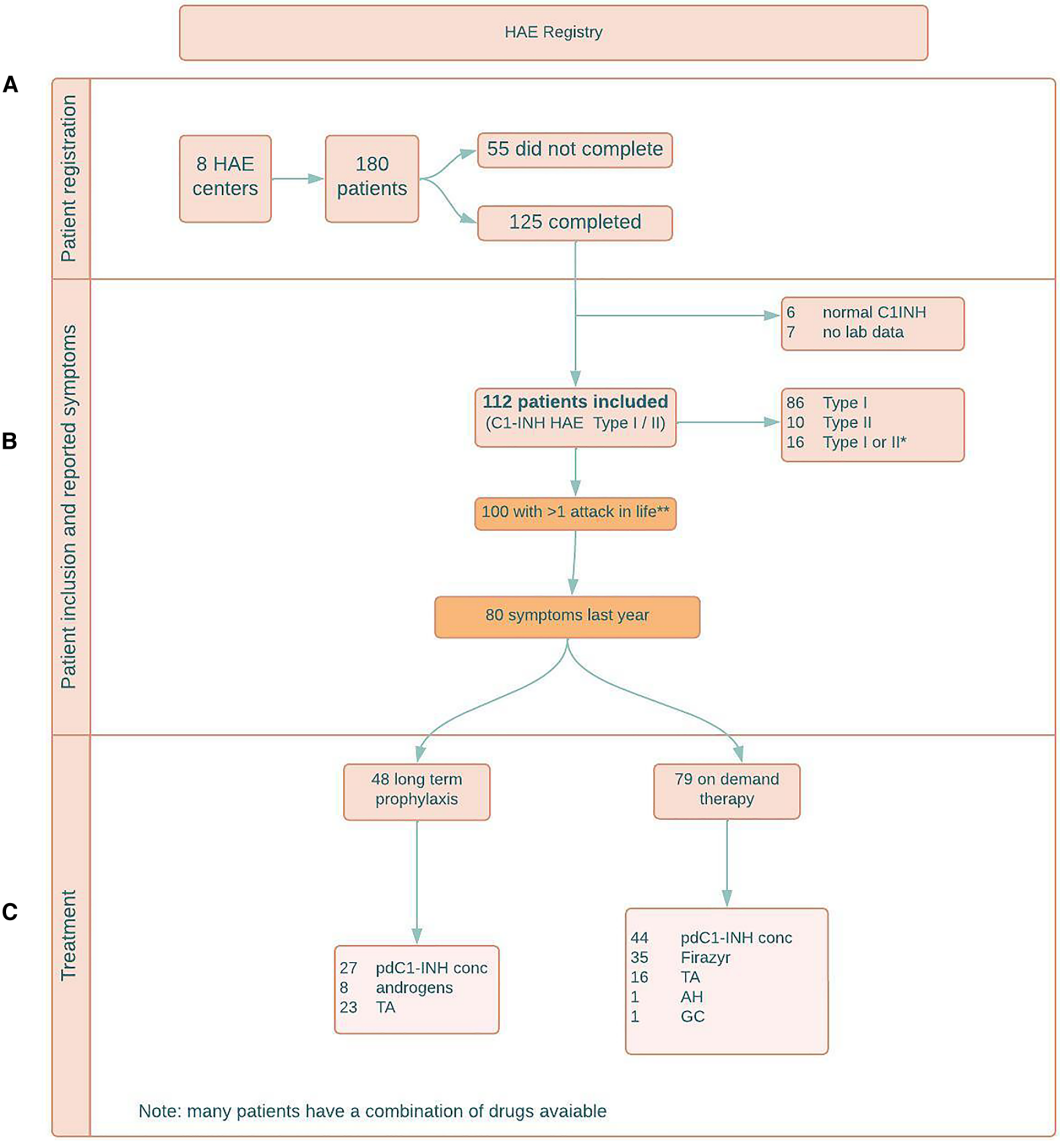
(**A**) patient registration; (**B**) patient inclusion and reported symptoms; (**C**) treatment. HAE = hereditary angioedema; y = years; C1INH = C1 esterase inhibitor; combi = combination of symptoms; C1-INH HAE = C1 esterase inhibitor deficiency causing HAE; pdC1-INH conc = plasma derived C1 esterase inhibitor concentrate; TA = tranexamic acid; AH = antihistamines; GC = glucocorticoids.

Registration of patients is shown in Figure 1A. By February 3rd 2022, a total of 125 patients (50 (40%) male, 75 (60%) female), completed the questionnaire.

Another 55 C1-INH-HAE patients who received the questionnaire did not answer or stated they were not willing to participate.

6/125 (4.8%) patients who filled out the questionnaire, were diagnosed with normal C1-INH-HAE. In 7/125 (5.6%) patients, the type of HAE was unknown (no lab data available). These patients were excluded.

Hence, as shown in Table 1 and Figure 1B, 112 patients with type I or type II C1-INH-HAE could be included.

**Table 1:** Data of included patients with type I and/or II HAE.

Included patients	
Total (*n*)	112
Type I HAE (*n*)	86
Type II HAE (*n*)	10
Unknown (Type I or II) (*n*)	16
Symptoms	
Age of onset (y) (median, range)	13.5 (0−57)
Diagnostic delay (y) (median, range)	7 (0–49)
Symptoms at least once/live (*n*)	100
Symptoms last year (*n*)	80
Prophylactic treatment	
Total (*n*)	48
pdC1-INH conc (*n*)	27
Androgens (*n*)	8
Tranexamic acid (*n*)	23
On demand treatment	
Total (*n*)	79
Firazyr	35
Tranexamic acid (*n*)	16
Antihistamines	1
Glucocorticoids	1

*n*, number; HAE, hereditary angioedema; y, years; pdC1-INH conc, plasma derived C1 esterase inhibitor concentrate; AH, antihistamines; GC, glucocorticoids.

Type I C1-INH-HAE was diagnosed in 86/112 (76.8%) patients, type II C1-INH-HAE was diagnosed in 10/112 (8.9%) patients. In 16/112 (14.3%) patients, C1-INH function was low, but additional antigenic C1-INH quantification was not performed, hence no distinction can be made between type I and type II.

### Symptoms

As shown in Figure 1B, 100/112 (89.3%) type I/II C1-INH-HAE patients experienced symptoms at least once in their lives.

The median age of onset of symptoms was 13.5 years (range 0–57 years). The median age at diagnosis was 20 years (range 0–71 years). The median time between onset and diagnosis was 7 years (range 0–49 years). Twelve patients who never experienced angioedema were diagnosed with the condition after a family member was affected (first degree relative). The diagnosis in these patients was based upon a low C1-INH function and/or low quantitative levels of C1-INH.

During the previous year, 80/112 patients experienced symptoms.

Of the 80 patients that experienced symptoms last year, the median number of attacks was 6 (range 1–60) and 29 patients visited the emergency department with an HAE attack at least once.

**Figure 2:**
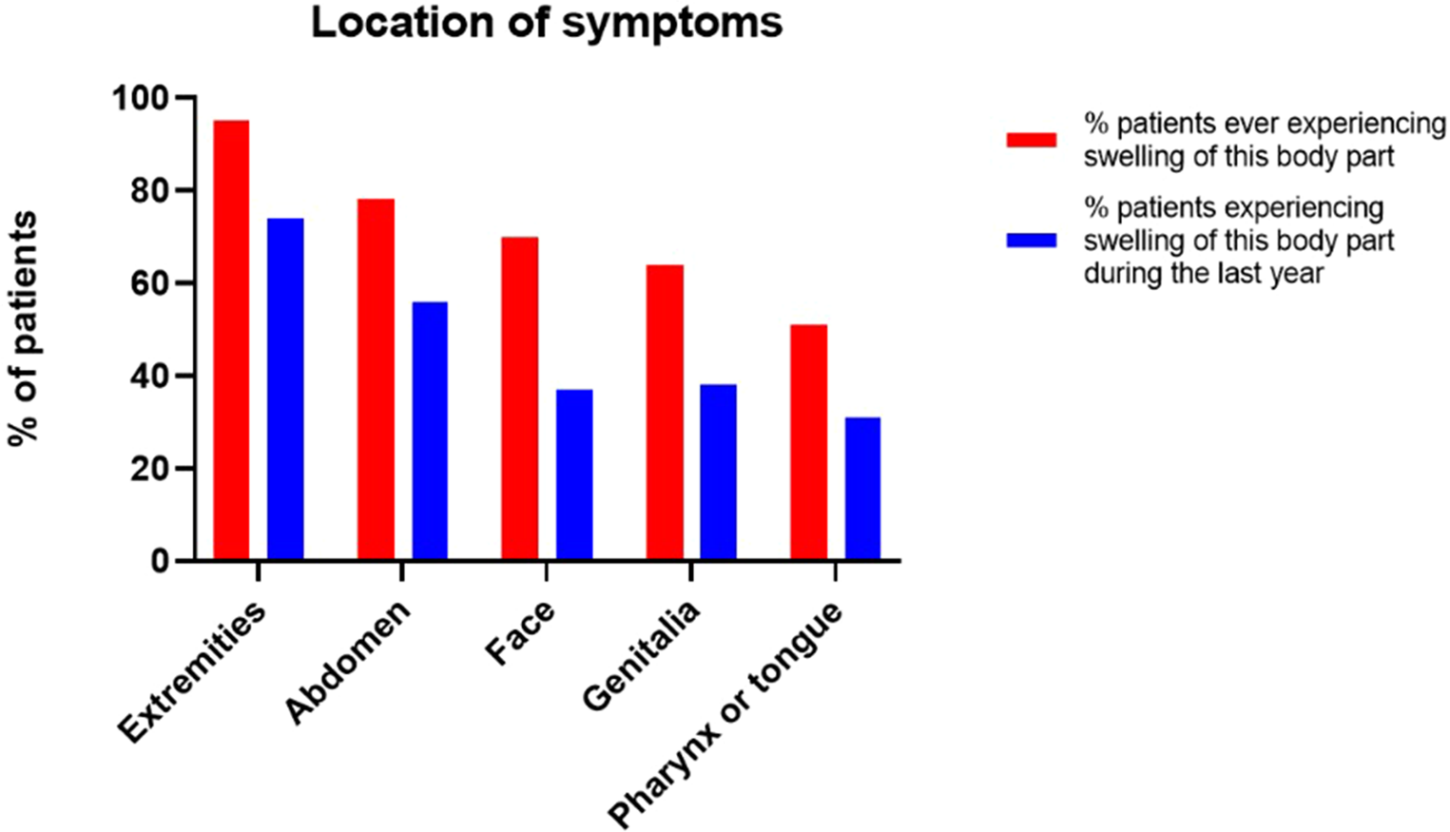
Location of symptoms.

As shown in Figure 2, the most reported symptoms were swelling of the extremities and abdominal symptoms (pain, nausea, vomiting, diarrhea, constipation), with respectively 95/100 (95%) and 78/100 (78%) patients that ever experienced symptoms, reporting to have experienced these at least once in their lives and respectively 74 (74%) and 56 (56%) patients during the last year.

70 (70%) patients experienced facial swelling at least once in their live, of whom 37 (37%) patients during the last year. Genital swelling was reported by 64 (64%) patients to have occurred at least once in their lives and by 38 (38%) patients during the last year.

51 (51%) patients experienced pharyngeal or tongue swelling at least once in their lives, of whom 31 (31%) patients during the last year.

**Figure 3:**
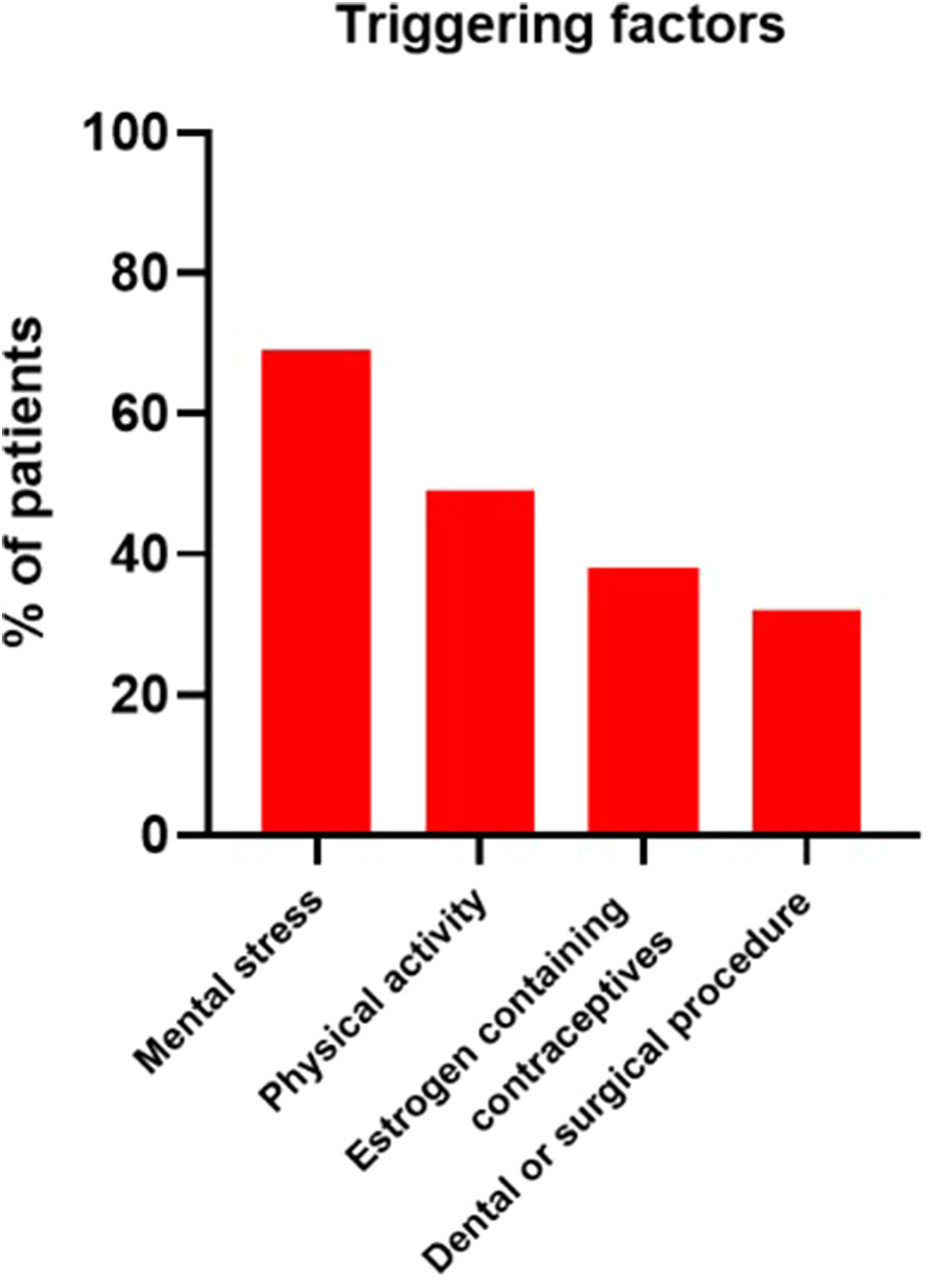
Four most frequently mentioned triggering factors. Other triggers included specific types of food, alcohol consumption, weather changes, viral infection and sleep deprivation.

As shown in Figure 3, the most common triggers were mental stress (69/100; 69%) and physical activity (49/100; 49%). 38 out of 63 (60.3%) adult women who ever reported symptoms, experienced more attacks whilst taking estrogen containing contraceptives. In 32 patients, an attack was triggered by a dental or surgical procedure.

### Sick leave

49/100 (49%) symptomatic patients, reported absenteeism of work/school during their last year.

The median number of absenteeism periods was 3 (range 1–20).

13 patients reported that their school/work environment were unsupportive of their situation.

35 patients missed out on activities they considered as important in their lives, because of HAE attacks (e.g. physical exercise, writing or travelling).

### Treatment

54 patients had used long term prophylactic therapy at some point in their lives and 48 patients were using long term prophylactic therapy at the time they responded to the questionnaire. This is 60% (48/80) of the 80 patients who experienced symptoms last year.

As shown in Figure 1C, the most common used long term prophylactic therapy was a human plasma-derived C1-esterase inhibitor concentrate (pdC1-INH concentrate), used by 27/48 (56.3%) patients. The median number of attacks during the last year in the patients taking a pdC1-INH concentrate was 5, compared to 3 in the patient group not on appropriate long-term prophylaxis.

8/48 (16.7%) patients used a 17-α-alkylated androgen as long term prophylactic therapy.

13/48 (27.1%) patients reported daily off-label use of tranexamic acid as only long term prophylactic therapy and another 10/48 (20.8%) patients used tranexamic acid in combination with pdC1-esterase INH concentrate or androgens.

Finally, 2 patients did not specify the long term prophylactic therapy they were actually taking.

As shown in Figure 1C, 79 (70.5%) patients reported that they had an on-demand treatment available in case of attacks; 44/79 (55.7%) in the form of pdC1-esterase INH concentrate, 35 (44.3%) in the form of the bradykinin 2 receptor antagonist icatibant and 5 of them had a combination of both available. Another 16 patients reported to use a pdC1-esterase INH concentrate or icatibant in combination with tranexamic acid, one patient in combination with antihistamines and another patient in combination with glucocorticosteroids.

Only 5/79 patients had no pdC1-esterase INH concentrate or icatibant available, 4 of them only had on-demand treatment in the form of transexamic acid, 1 patient in the form of H1-antihistamines.

2/79 patients reported to take butylscopolamine for abdominal cramping.

8/79 patients had an epinephrine auto-injector available.

Many patients had multiple drugs available, hence the total number of drugs does not add up to the total number of patients.

## Discussion

C1-INH HAE is a rare hereditary disease characterized by recurrent subepithelial swelling of sudden onset due to the generation of the highly potent vasodilator bradykinin. This is the first nationwide survey of C1-INH HAE in Belgium. 112 patients with HAE Type I and II could be included. Moreover, we know of 55 C1-INH-HAE patients who were sent, but did regrettably not complete the questionnaire.

This gives a minimum prevalence of 1.56/100.000 in Belgium, largely similar to other European countries like Sweden (1.54/100.000), Denmark (1.41/100.000), Italy (1.54/100.000) and Spain (1.09/100.000) (8, 10–12). However, we believe for this to be an underestimation. Patients were selected to participate through the reference centers, and a lack of clinical recognition by general physicians and limited referral to these centers, might make the real prevalence of HAE in Belgium to be higher.

Despite HAE being a rare disease, diagnostic error and delay comes with serious consequences such as reduced quality of life and, in case of laryngeal and/or tracheal angioedema, even risk of rapid asphyxiation and death (19, 20).

Hence, correct management and knowledge of the current guidelines, is of utmost importance. Even though the patients were included through HAE reference centres, the participating physicians reported for their patients to consult only once every one to two year(s), in the mean time being followed by their general physician. Non-specialist physicians might not be aware of the guidelines of such a rare pathology.

It is evident that our data disclose many areas needing urgent improvement in terms of recognition, disease management and correct treatment.

First, we report a median diagnostic delay of 7 years. This once again underlines the importance to raise more awareness among general physicians and specialists.

Second, in many patients, the disease was not properly controlled. 51% of patients had experienced pharyngeal or tongue swelling and 78% had experienced abdominal symptoms, both known to cause an important reduction in quality of life. Moreover, 70% had experienced facial swelling, which can be stigmatizing and a cause of a low self-esteem. Nearly half the patients reported absenteeism during their last year at school or work.

A limitation of this study is that the quality of life was only assessed informally, by rating the number of attacks, the type of symptoms and absenteeism. However, this also depicts an important gap in our current management of HAE. After all, current guidelines recommend that a formal quality of life assessment is performed at least yearly.

Moreover, we report the use of non-recommended treatment options, potentially explaining the high disease burden. As recommended in the WAO guidelines, plasma-derived C1 inhibitor should be a first-line choice. However, still almost half of patients on long term prophylactic therapy, reported the daily off-label use of tranexamic acid. Neither the new revised guidelines, nor the 2017 guidelines, in use at the start of this study, recommend antifibrinolytics for long-term prophylaxis. The reason for this probably relates to the fact that many patients are primarily followed by non-specialist physicians who might continue (off-label) treatments that are not standard care anymore. Moreover, patients might be reluctant to start (seemingly invasive) intravenous or subcutaneous treatment and patients are often not compliant to the recommended usage frequency.

As was mentioned in the results, the median number of attacks during the last year was higher in the patients taking a pdC1-INH concentrate compared to the patients not on appropriate long-term prophylaxis. However, this is assumed to be biased by the fact that patients experiencing more severe symptoms, would likely be considered sooner for prophylactic treatment. A follow-up study regarding the longitudinal effect of this treatment on the patients’ symptoms, could be interesting for future research.

In the new guidelines, the use of a monoclonal antibody kallikrein inhibitor is also recommended as first-line prophylaxis. However, in Belgium, reimbursement for this drug was only approved in June 2022.

A final gap we could identify in the management of Belgian HAE patients, it the fact that only 70.5% of patients have on demand treatment available, while guidelines recommend this for all patients.

However, these numbers are high compared to other European studies. In Sweden, only 27% of patients had a recommended on-demand treatment option. It is of note that icatibant was not available at the time of the Swedish survey (11).

Almost all of these patients report to use pdC1-INH concentrate and/or icatibant. However, there is still room for improvement, since 5 patients reported tranexamic acid or H1-antihistamines to be the only treatment they had available during an acute attack, while neither of them should be considered on-demand therapy.

In conclusion, this paper provides the opportunity to emphasize the recently updated guidelines (2) for the management of HAE. We believe that our study provides a representation overview of the type I and II C1-INH-HAE patients in Belgium. From our data, it emerges that many physicians seem not to be up to date about the current guidelines for the treatment of HAE (the study was performed in H with the highest levels of expertise/please formulate differently). Hence the importance of this study and the dissemination of these data, which we believe will raise awareness about the disease and will benefit the recognition and management.

## Data Availability

The raw data supporting the conclusions of this article will be made available by the authors, without undue reservation.

## References

[1] Longhurst H Cicardi M. Hereditary angio-oedema. Lancet. (2012) 379(9814):474–81. 10.1016/S0140-6736(11)60935-5 22305226

[2] Maurer M Magerl M Betschel S Aberer W Ansotegui IJ Aygören-Pürsün E The international WAO/EAACI guideline for the management of hereditary angioedema-the 2021 revision and update. Allergy. (2022) 77(7):1961–90. 10.1111/all.15214. Epub 2022 Feb 3. PMID: . 35006617

[3] Farkas H Martinez-Saguer I Bork K Bowen T Craig T Frank M International consensus on the diagnosis and management of pediatric patients with hereditary angioedema with C1 inhibitor deficiency. Allergy. (2017) 72(2):300–13. 10.1111/all.13001 27503784 PMC5248622

[4] Ebo DG Van Gasse AL Sabato V Bartholomeus E Reyniers E Vanbellinghen JF Hereditary angioedema in 2 sisters due to paternal gonadal mosaicism. J Allergy Clin Immunol Pract. (2018) 6(1):277–279.e1. 10.1016/j.jaip.2017.07.002 28888847

[5] Ebo DG Blaumeiser B Kooy FR Beckers S Van Gasse AL Saerens M Association of hereditary angioedema type 1 with developmental anomalies due to a large and unusual de novo pericentromeric rearrangement of chromosome 11 spanning the entire C1 inhibitor gene (SERPING1). J Allergy Clin Immunol Pract. (2019) 7(4):1352–1354.e3. 10.1016/j.jaip.2018.10.005 30336291

[6] Bork K Machnig T Wulff K Witzke G Prusty S Hardt J. Clinical features of genetically characterized types of hereditary angioedema with normal C1 inhibitor: a systematic review of qualitative evidence. Orphanet J Rare Dis. (2020) 15(1):289. 10.1186/s13023-020-01570-x 33059692 PMC7559394

[7] Sharma J Jindal AK Banday AZ Kaur A Rawat A Singh S Pathophysiology of hereditary angioedema (HAE) beyond the SERPING1 gene. Clin Rev Allergy Immunol. (2021) 60(3):305–15. 10.1007/s12016-021-08835-8 33442779

[8] Roche O Blanch A Caballero T Sastre N Callejo D López-Trascasa M Hereditary angioedema due to C1 inhibitor deficiency: patient registry and approach to the prevalence in Spain. Ann Allergy Asthma Immunol. (2005) 94(4):498–503. 10.1016/S1081-1206(10)61121-0 15875532

[9] Johnsrud I Kulseth MA Rødningen OK Landrø L Helsing P Waage Nielsen E A nationwide study of Norwegian patients with hereditary angioedema with C1 inhibitor deficiency identified six novel mutations in SERPING1. PLoS One. (2015) 10(7):e0131637. 10.1371/journal.pone.0131637 26154504 PMC4496036

[10] Bygum A. Hereditary angio-oedema in Denmark: a nationwide survey. Br J Dermatol. (2009) 161(5):1153–8. 10.1111/j.1365-2133.2009.09366.x 19709101

[11] Nordenfelt P Nilsson M Björkander J Mallbris L Lindfors A Wahlgren CF Hereditary angioedema in Swedish adults: report from the national cohort. Acta Derm Venereol. (2016) 96(4):540–5. 10.2340/00015555-2274 26540175

[12] Zanichelli A Arcoleo F Barca MP Borrelli P Bova M Cancian M A nationwide survey of hereditary angioedema due to C1 inhibitor deficiency in Italy. Orphanet J Rare Dis. (2015) 10:11. 10.1186/s13023-015-0233-x 25758562 PMC4333895

[13] Psarros F Koutsostathis N Farmaki E Speletas MG Germenis AE. Hereditary angioedema in Greece: the first results of the Greek hereditary angioedema registry. Int Arch Allergy Immunol. (2014) 164(4):326–32. 10.1159/000366276 25277223

[14] Sobotkova M Zachova R Hakl R Kuklinek P Kralickova P Krcmova I Acquired angioedema with C1 inhibitor deficiency: occurrence, clinical features, and management: a nationwide retrospective study in the Czech republic patients. Int Arch Allergy Immunol. (2021) 182(7):642–9. 10.1159/000512933 33472202 PMC8315685

[15] Gabos G Nadasan V Mihaly E Dobru D Hereditary angioedema due to C1-inhibitor deficiency in Romania: first national study, diagnostic and treatment challenges. Iran J Immunol. (2020) 17(3):226–35. 10.22034/iji.2020.85416.1709. PMID: . 32996899

[16] Grivčeva-Panovska V Košnik M Korošec P Andrejević S Karadža-Lapić L Rijavec M Hereditary angioedema due to C1-inhibitor deficiency in Macedonia: clinical characteristics, novel SERPING1 mutations and genetic factors modifying the clinical phenotype. Ann Med. (2018) 50(3):269–76. 10.1080/07853890.2018.1449959 29513108

[17] Rijavec M Korošec P Šilar M Zidarn M Miljković J Košnik M Hereditary angioedema nationwide study in Slovenia reveals four novel mutations in SERPING1 gene. PLoS One. (2013) 8(2):e56712. 10.1371/journal.pone.0056712 23437219 PMC3577750

[18] Guryanova I Suffritti C Parolin D Zanichelli A Ishchanka N Polyakova E Hereditary angioedema due to C1 inhibitor deficiency in Belarus: epidemiology, access to diagnosis and seven novel mutations in SERPING1 gene. Clin Mol Allergy. (2021) 19(1):3. 10.1186/s12948-021-00141-0 33827715 PMC8028818

[19] Bork K Anderson JT Caballero T Craig T Johnston DT Li HH Assessment and management of disease burden and quality of life in patients with hereditary angioedema: a consensus report. Allergy Asthma Clin Immunol. (2021) 17(1):40. 10.1186/s13223-021-00537-2 33875020 PMC8056543

[20] Lee EY Hsieh J Borici-Mazi R Caballero T Kanani A Lacuesta G Quality of life in patients with hereditary angioedema in Canada. Ann Allergy Asthma Immunol. (2021) 126(4):394–400.e3. 10.1016/j.anai.2021.01.002 33450396

